# Comparison of proteomic landscape of extracellular vesicles in pleural effusions isolated by three strategies

**DOI:** 10.3389/fbioe.2023.1108952

**Published:** 2023-04-12

**Authors:** Xue Yao, Baixue Liao, Feng Chen, Lüye Liu, Kaiwen Wu, Yaying Hao, Yanping Li, Yuebin Wang, Ruiling Fan, Jun Yin, Lei Liu, Yuanbiao Guo

**Affiliations:** ^1^ School of Medicine, Southwest Jiaotong University, The Affiliated Hospital of Southwest Jiaotong University, The Third People’s Hospital of Chengdu, Chengdu, China; ^2^ Department of Respiratory, The Third People’s Hospital of Chengdu, The Affiliated Hospital of Southwest Jiaotong University, Chengdu, China; ^3^ Medical Research Center, The Third People’s Hospital of Chengdu, The Affiliated Hospital of Southwest Jiaotong University, Chengdu, China; ^4^ School of Pharmacy, North Sichuan Medical College, Nanchong, China

**Keywords:** extracellular vesicles, pleural effusion, ultracentrifugation, size exclusion chromatography, density gradient ultracentrifugation, proteomics

## Abstract

Extracellular vesicles (EVs) derived from pleural effusion (PE) is emerging as disease biomarkers. However, the methods for isolation of EVs from PE (pEVs) were rarely studied. In our study, three methods for isolating pEVs of lung cancer patients were compared, including ultracentrifugation (UC), a combination of UC and size exclusion chromatography (UC-SEC) and a combination of UC and density gradient ultracentrifugation (UC-DGU). The subpopulation of pEVs was identified by nanoparticle tracking analysis (NTA), transmission electron microscopy (TEM), Western blotting (WB) and nano-flow cytometry (nFCM). Additionally, the proteomic landscape of pEVs was analyzed by Label-free proteomics. The results showed that, compared with UC and UC-DGU, the UC-SEC method separated pEVs with the highest purity. In the proteomic analysis, on average, 1595 proteins were identified in the pEVs isolated by UC-SEC, much more than pEVs isolated by UC (1222) or UC-DGU (807). Furthermore, approximately 90% of identified proteins in each method were found in the EVs public database ExoCarta. Consistent with this, GO annotation indicated that the core proteins identified in each method were mainly enriched in “extracellular exosome.” Many of the top 100 proteins with high expression in each method were suggested as protein markers to validate the presence of EVs in the MISEV2018 guidelines. In addition, combined with lung tissue-specific proteins and vesicular membrane proteins, we screened out and validated several novel protein markers (CD11C, HLA DPA1 and HLA DRB1), which were enriched in pEVs rather than in plasma EVs. In conclusion, our study shows that the method of UC-SEC could significantly improve the purity of EVs and the performance of mass spectrometry-based proteomic profiling in analyzing pEVs. The exosomal proteins CD11C, HLA DPA1 and HLA DRB1 may act as potential markers of pEVs. The proteomic analysis of pEVs provides important information and new ideas for studying diseases complicated with PE.

## Graphical Abstract

**Figure:**
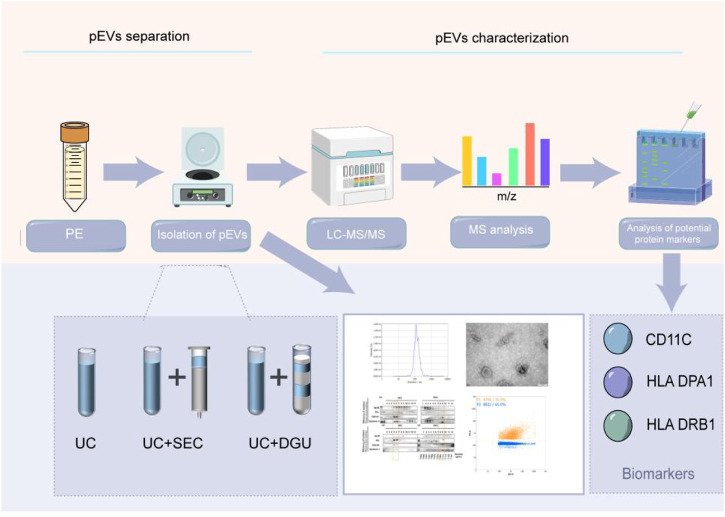
In our study, three methods were used for isolating extracellular vesicles derived from pleural effusion (pEVs), including ultracentrifugation (UC), a combination of UC and size exclusion chromatography (UC-SEC) and a combination of UC and density gradient ultracentrifugation (UC-DGU), and followed with proteomics analysis. Our results show that the method of UC-SEC could significantly improve the purity of pEVs and improve the performance of proteomic analysis of pEVs. The exosomal proteins CD11C, HLA DPA1 and HLA DRB1 may act as potential markers of pEVs.

## Introduction

Pleural effusion (PE) is a common complication of the body when responding to systemic diseases such as cancer, infection, or inflammation (KJ et al., 2019; Watabe et al., 2020; Ai et al., 2022). PE is divided into benign pleural effusion (BPE) and malignant pleural effusion (MPE) according to the type of disease. Lung cancer invading the pleura could lead to the production of MPE, which symptoms is very similar to pneumonia, so it is important to improve the differential diagnosis of lung cancer and other non-malignant lung diseases (Egan et al., 2014). The cellular composition and biochemical indices of PE are commonly used in standard clinical laboratories. However, pleural biopsy often results in delayed or missed diagnosis because a lot of patients with a low load of tumor cells in pleural fluid. Although such clinical tests typically achieve 100% specificity, their average sensitivity to the diagnosis of malignancy is only 51.3% (AM et al., 2020). Molecularly targeted therapies for lung cancer have improved patient survival compared with traditional chemotherapy. Nevertheless, obtaining tumor tissue for molecular analysis was often difficult and sometimes brings risks to the patients (Tan and Tan, 2022).

Extracellular vesicles (EVs) are bilayer vesicles with a diameter of 30–200 nm (Théry et al., 2018; Zhang et al., 2019; Brambilla et al., 2021). EVs contain proteins, nucleic acids, and metabolites (Skotland et al., 2019), play an important role in intracellular and intercellular communication, and participate in various physiological and pathological processes (Théry et al., 2002; Palazzolo et al., 2020). EVs are found in most kinds of body fluids, such as blood (Karimi et al., 2018), saliva (Yu et al., 2022), urine (Li and Yang, 2022), cerebrospinal fluid (Kong et al., 2018), pleural effusion (Luo et al., 2020), ascites (Pascual-Antón et al., 2021), and so on (Dixon et al., 2018; Kaddour et al., 2020), which are considered to be potential biomarkers of many diseases, such as cancer, neurological disorders, diabetes and kidney disease (Ashrafizadeh et al., 2022; Thongboonkerd and Kanlaya, 2022; Zhou et al., 2022). The contents of EVs secreted from cells or organs of patients were different from the healthy population (Jin et al., 2017; Lin et al., 2018; Chen et al., 2020). Like other biological fluids, EVs in addition to cells and other macromolecules in PE are potential carriers for biomarkers of lung disease (Li et al., 2016; Xiao et al., 2019; Javadi et al., 2021). There is new evidence that EVs are involved in lung cancer progression, including angiogenesis, epithelial interstitial transformation, immune system suppression, metastasis, drug resistance, and so on (Saviana et al., 2021; Li et al., 2022; Zhou et al., 2022). Because PE is anatomically close to cancerous tissue, pEVs may act with greater potential in the diagnosis of lung diseases (Antonopoulos et al., 2021).

Although EVs are of interest as potential biomarkers, their basic research and clinical applications are greatly limited due to the time-consuming, laborious and inefficient isolation and purification processes of EVs (Martins et al., 2022; Ströhle et al., 2022). The origin and separation conditions will affect the purity of EVs (Ibsen et al., 2017; Dong et al., 2020; Guo et al., 2021). For example, serum and plasma contain a large number of non-EV lipid structures (low/very low/high-density lipoprotein), milk is filled with fat-containing vesicles and urine contains urine regulatory protein (Tamm Horsfall protein) and bronchoalveolar lavage fluid contain surfactant (Fernández-Llama et al., 2010; Ibsen et al., 2017; Yang et al., 2019; Sedykh et al., 2020), all of which will be co-separated in varying degrees from EVs. Therefore, different separation strategies may need to be adopted in different situations. One of the main challenges of EVs separation is the elimination of nanoscale contaminants (Royo et al., 2020; Brambilla et al., 2021). ApoB is co-isolated as a contaminant in EVs from PE, which may hinder the efficient separation of high-purity EVs (Valdés et al., 2010). Therefore, additional purification steps are usually required before analyzing pEVs.

Ultracentrifugation is currently the main technology for separating EVs (Lobb et al., 2015; Li M. et al., 2021; Chhoy et al., 2021; Staubach et al., 2021). It is widely used in the discovery of EV-related biomarkers, but it is unable to remove rich lipoprotein particles, such as high-density lipoprotein (HDL), which may affect downstream mass spectrometry analysis of proteins (An et al., 2018; Liu et al., 2022). Size exclusion chromatography (SEC) has long been used to separate particles based on their size (Gordon et al., 1943; Gordon et al., 1944; Kaddour et al., 2021; Yang et al., 2021). Although the EVs isolated by the SEC are relatively pure and the method is simple, they cannot distinguish EVs from the particles with similar size and microbubbles (Sidhom et al., 2020). So, despite its long history, affordability, and widespread availability, SEC is still in its infancy in the EV field (Kaddour et al., 2021). Density gradient ultracentrifugation (DGU) is to use of a certain medium in the centrifuge tube to form a continuous or discontinuous density gradient, the sample to be separated is placed on the top or bottom of the medium, then the sample was separated through the action of gravity or centrifugal force field. DGU can isolate relatively pure EVs, but it also has some disadvantages, such as more contamination of lipoprotein particles in the final product (Yuana et al., 2014; Sidhom et al., 2020), generation of EVs aggregates, and time-consuming. There are also other separation methods, such as precipitation of polyethylene glycol groups (Quan et al., 2019), affinity capture (Filipović et al., 2022), and membrane affinity (Liang et al., 2022), but the efficiency is limited. Since it is difficult to balance the yield and purity with only one method, many researchers have proposed combining different separation methods and proved that it can effectively improve the efficiency of separation (Koh et al., 2018; Ryu et al., 2020; Zhang et al., 2020).

To evaluate the combinatorial strategies in the separation of pEVs, we compared the efficiency of UC, UC-SEC and UC-DGU methods by nanoparticle tracking analysis (NTA), transmission electron microscopy (TEM), Western blotting (WB) and nano-flow cytometry (nFCM). In addition, the proteomic landscape of pEVs isolated by different methods was analyzed by LC-MS proteomic analysis. By evaluating the yield, purity and protein characteristics of pEVs extracted by different methods, experimental evidence was provided for separating high-quality EVs from pleural effusion for proteomic analysis. Moreover, several novel potential markers of pEVs (CD11C, HLA DPA1 and HLA DRB1) may be identified.

PE is a bodily fluid that has received relatively less attention in terms of EVs separation techniques and associated markers, despite its widespread availability. The source of the sample affects the purity of the EVs, so different body fluids may require different isolation strategies (Dong et al., 2020; Pascual-Antón et al., 2021). Our study provides a new idea for further understanding of the separation method of pEVs and lays a foundation for further proteomic analysis based on pEVs.

## Materials and methods

### Patients and samples collection

Blood samples were collected from 7 patients of non-small cell lung cancer (NSCLC) with malignant pleural effusion (MPE), and PE samples were collected from 16 patients of NSCLC with MPE and 6 patients with bacterial pneumonia with benign pleural effusion (BPE). All patients were diagnosed at the Third People’s Hospital of Chengdu between June 2020 and June 2022. Among these, PE samples from 3 patients with MPE were used for comparison of isolation methods, Blood samples from 7 patients with MPE and PE samples from 13 patients with MPE and 6 cases with BPE were used for subsequent validation. The present study was approved by the Ethics Committee of the Third People’s Hospital of Chengdu and signed informed consent was obtained from the patients. All patients with lung cancer were newly diagnosed without receiving any chemotherapy or radiation therapy and histologically confirmed by two different pathologists.

For PE samples collection, PE was freshly collected and aliquoted using Falcon™ 50 mL conical centrifuge tubes and stored at −80 °C. For blood samples collection, the fasting venous blood of the subjects was collected by EDTA anticoagulant tube. After that, the blood samples were processed to deplete platelets and blood cells by two-step centrifugation at 2,500 × g for 15 min at room temperature. Then the plasma was aliquoted and stored at −80 °C for further study.

### Isolation of pEVs

The pEVs were isolated from 200 mL pleural effusion by UC, UC-SEC and UC-DGU. To separate cells, cellular debris, and microvesicles from PE, the supernatant was centrifuged at 300 × g for 10 min, 2,000 × g for 30 min and 10,000 × g for 60 min at 4°C. Then, the supernatant was filtered through 0.22 μm filters and centrifuged at 110,000×g for 70 min at 4°C (Beckman, L-100XP, USA). The pellet was resuspended with PBS, which was considered crude pEVs, and the crude resuspending was used for the following purification steps.

### Ultracentrifugation (UC)

For the UC method, the crude pEVs was centrifuged at 110,000×g for 70 min at 4°C, then resuspended in PBS for characterization and LC-MS analysis. For the validation experiment, EVs from 30 mL pleural effusion and 2 mL plasma samples were obtained by UC method.

### Size exclusion chromatography (SEC)

For SEC method, qEV original size exclusion column (IZON Science, Christchurch, NZ) was used according to the instructions of the manufacturer. Briefly, columns were equilibrated with 20 mL of PBS before use. Then, 500 μL of the crude pEVs sample was pipetted onto the column and eluted with PBS. The eluent was collected in 13 sequential fractions with 1 mL for each fraction and stored at − 80 °C for subsequent study.

### Density gradient ultracentrifugation (DGU)

For DGU method, fractionation of pEVs was accomplished using OptiPrep density gradient medium (60% iodixanol solution, Sigma-Aldrich, St. Louis, USA). Firstly, PBS was mixed with OptiPrep at a volume ratio of 1:5 to prepare the working solution (the working solution concentration is 50%). Then, iodixanol solutions of 40%, 20%, 10% and 5% (wt/vol) with different concentrations were prepared with the working solution. By adding 50 µL 0.4% (wt/vol) Trypan Blue solution to 20% and 5% (wt/vol) iodixanol solutions, clear differences between the layers can be easily seen. Then, the tube was slowly tilted to 70° and each iodixanol solution was carefully transferred onto the surface of the liquid. 3.2 mL of 40%, 20% and 10% (wt/vol) iodixanol solutions were layered and followed by 2.2 mL of 5% (wt/vol) iodixanol solutions, which formed a discontinuous gradient of iodixanol. 1 mL of the crude pEVs sample was carefully transferred onto the surface of the liquid, and then centrifuged at 110,000×g for 16 h at 4°C. After that, the layers are no longer clear between the concentration gradients. Each fraction was collected in 800 uL from the top of the gradient medium by P100 pipettes and stored at − 80 °C for subsequent analysis. The final density of the collected fractions can be determined by making a standard curve of the absorbance values at 340 nm of 1:1 aqueous dilutions of 5, 10, 20% and 40% iodixanol.

### Characterization of pEVs

#### Nanoparticle tracking analysis (NTA)

The particle size and quantity of extracellular vesicles were measured by Nanoparticle tracking analysis (NTA) with ZetaView (Particle Metrix, Meerbusch, Germany). The sample concentration was adjusted from 50–200 particles per frame by dilution from 1:100 to 1:10,000 in ultrapure water. The unique analysis parameter was set as: Sensitivity 70, Shutter 70, Minimum brightness: 20, Maximum area: 1000, Minimum Area: 5, Laser Wavelength: 488 nm. Using ZetaView (version 8.05.11), each measurement scan was performed at 11 different positions.

#### Transmission electron microscopy (TEM)

Transmission electron microscopy (TEM) (JEOL, JEM-1400, Japan) was used to visualize extracellular vesicles. Freshly isolated extracellular vesicles were placed on a copper grid and kept at room temperature for 5 minutes, then stained with 2% (v/v) uranyl acetate and examined immediately after staining.

### Bicinchoninic acid (BCA) assay

According to the manufacturer’s instructions, the protein concentration was determined using the BCA assay (Thermo Scientific, IL, USA).

### Western blotting (WB)

10% SDS-PAGE was used to load proteins of equal volume and amount. The purpose of loading equal volumes of fractions was to assess the peak fraction and determine the efficiency of the EVs isolation among eluted fractions. Then the proteins were transferred onto the PVDF membrane (Millipore, MA, USA). The membranes were blocked with 5% non-fat dry milk in TBST for 1 h at room temperature, followed by primary antibodies incubation overnight at 4 °C. The following primary antibodies were used at a 1: 1000 dilution: ApoB (Proteintech, Wuhan, China), Alix (Cell Signaling Tech, MA, USA), TSG101 (Abcam, Cambridge, United Kingdom), Syntenin-1 (Proteintech, Wuhan, China), HLA-DPA1 (PTM BIO, Hangzhou, China), HLA-DRA (PTM BIO, Hangzhou, China), HLA-DRB1(PTM BIO, Hangzhou, China), HLA-DRB5(PTM BIO, Hangzhou, China), ITGAX (Proteintech, Wuhan, China), MRC1(Proteintech, Wuhan, China) antibodies. The HRP-conjugated anti-mouse IgG or HRP-conjugated anti-rabbit IgG (Abcam, Cambridge, United Kingdom) was used as the secondary antibody at a 1: 5000 dilution for 90 min at room temperature. Enhanced chemiluminescence reagent (Millipore, MA, USA) was then used for the visualization of the membranes.

### Nano-flow cytometry (nFCM) analysis

The pEVs samples were analyzed for particle concentration, size distribution, and phenotyping of surface protein makers by the nFCM (NanoFCM Inc., Xiamen, China) (Tian et al., 2018). Briefly, 20 μL of PE-conjugated mouse anti-human CD9 antibody, PE-conjugated mouse anti-human CD63 antibody, PE-conjugated mouse anti-human CD81 antibody and PE-conjugated mouse IgG (BD Biosciences, San Jose, USA) was added into each 100 μL pEVs sample. The mixture was incubated at 37°C for 30 min and then washed with 13 mL PBS by ultracentrifugation at 110,000 × g for 70 min at 4 °C. The pellet was resuspended in 100 μL PBS for analysis. The nFCM analysis used two single photon counting avalanche photodiodes (APDs) to detect individual particle side scatter (SSC) and fluorescence simultaneously. To calibrate the instrument, 200 nm PE and AF488 fluorophore-conjugated polystyrene beads were used for particle concentration and Silica Nanosphere Cocktail (NanoFCM Inc., Xiamen, China) for particle size distribution. The detector recorded particles passing by during a 1-min interval in each test. Each sample was diluted to reach a particle count within the optimal range of 2000–12,000 particles per minute. NanoFCM software (NanoFCM Profession V2.0) was used to convert flow rate and side scattering intensity to vesicle concentration and size.

### Label-free quantitative proteomics

#### Protein extraction and digestion

The samples were subjected to sonication for a duration of 3 minutes with the assistance of a high-intensity ultrasonic processor (Scientz) while being kept on ice. The fragments that persisted were eliminated by performing centrifugation at 4 °C and 12,000 g for a duration of 10 minutes. The supernatant was obtained and quantified by BCA kit (Beyotime, Shanghai, China).

Each sample was enzymatically hydrolyzed with 30 ug protein. In preparation for digestion, the protein solution was reduced with 5 mM dithiothreitol for 30 min at 56 °C and alkylated with 11 mM iodoacetamide for 15 min at room temperature in darkness. The protein sample was then diluted by adding 200 mM TEAB to urea concentration of less than 2 M. Finally, trypsin was added at 1:50 trypsin-to-protein mass ratio for the first digestion overnight and 1:100 trypsin-to-protein mass ratio for a second 4-h digestion. Finally, the peptides were desalted by Strata X SPE column. After protease digestion, the peptides were also quantified, and 500 ng peptides were detected.

### LC-MS/MS

In solution A (0.1% formic acid, 2% acetonitrile in water), the tryptic peptides were dissolved and loaded directly onto a reversed-phase column (25 cm long, 75/100 mm in diameter). Label-free proteomics separated peptides with a gradient from 6% to 24% solvent B (0.1% formic acid in acetonitrile) over 70 min, 24%–35% in 14 min, and climbing to 80% in 3 min then holding at it for the last 3 min, all at a constant flow rate of 450 nL/min on a nanoElute UHPLC system (Bruker Daltonics).

Using a capillary source, peptides were mass spectrometically analyzed using timsTOF Pro (Bruker Daltonics). The voltage applied to the electrospray was 1.60 KV. A TOF detector was used to analyze precursors and fragments, with a range of 100–1700 m/z for the MS/MS scan. In parallel accumulation serial fragmentation mode (PASEF), the timsTOF Pro was operated. For fragmentation, precursors with charge states 0 to 5 were selected, and 10 PASEF-MS/MS scans were acquired per cycle. Dynamic exclusion was set to 30 s.

### Data analysis

The raw data was processed with the search engine MaxQuant (v.1.6.15.0). The following search parameters have been established: The database utilized is Homo_sapiens_9606_SP_20210721.fasta, containing a total of 20,387 sequences. In order to calculate the false positive rate (FDR) resulting from random matching, an anti-library has been added. Additionally, a common contamination library has been included within the database to mitigate the impact of contaminating proteins on the identification outcomes. The Trypsin/P enzyme digestion protocol was established with a limit of 2 missing cleavage sites and a minimum peptide length of 7 amino acid residues. Additionally, a restriction of a maximum of 5 peptide modifications was applied. In the first and main searches, the mass error tolerance for primary parent ions was established at 20 ppm, while for secondary fragment ions it was set at it. The fixed modification of Cysteine alkylated Carbamidomethyl (C) was established, while Methionine oxidation and N-terminal protein acetylation were designated as the modified modifications. The rate of false discovery for protein and PSM identification was fixed at 1%.

To obtain high-quality analysis results, search database analysis results need to be further filtered. The accuracy of FDR was set at 1% at the three levels of the spectrogram, peptide segment, and protein. For qualitative protein analysis, at least one unique peptide needs to be included. For protein quantification, at least two unique peptides need to be included.

### Enrichment of gene ontology analysis

According to GO annotation, proteins fall into three categories: cellular compartment, biological process, and molecular function. Two-tailed Fisher’s exact tests were conducted against all identified proteins in each category to determine whether the core proteins were enriched. The GO analysis was performed by FunRich (Version 3.1.3). There is significance for the GO with a corrected *p*-value of 0.05.

### Statistical analysis

Statistical analysis was performed using GraphPad Prism 8. All experiments were conducted at least three times. Student's t-tests were used to identify differences between two groups. *p* values < 0.05 was considered as significant difference.

## Results

### The isolation method impacts the yield and purity of pEVs

In order to search for the method suitable for pEVs separation, three methods (UC, UC-SEC and UC-DGU) were used in the present study.

**FIGURE 1:**
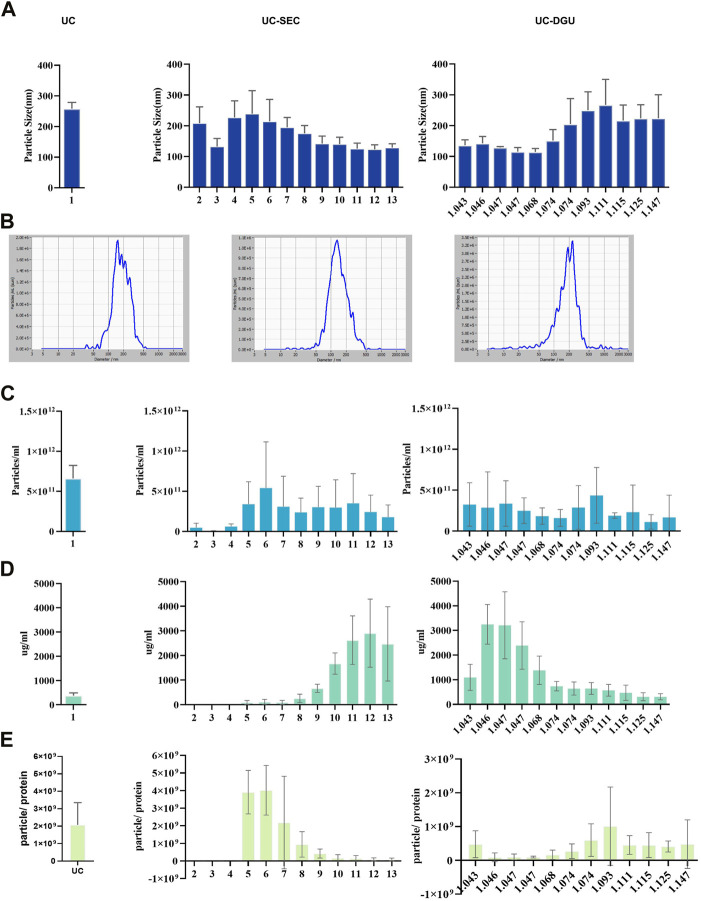
The yield and purity of pEVs. **(A)** The size distribution of pEVs separated by three methods were measured using nanoparticle tracking analysis (NTA). **(B)** Representative particle size distribution plots of pEVs separated by three methods were measured using NTA. **(C)** The concentration of pEVs particles separated by three methods was measured using NTA. **(D)** The protein concentration of isolated particles by three methods was measured by BCA assay. **(E)** The particle number/protein (ug) ratio of pEVs for three methods.

As a result, the pEVs have been successfully isolated by all three methods. However, there was a dramatic difference in the characteristic of pEVs particles among three methods. First, the particle sizes were measured by NTA and the diameters of pEVs isolated by three methods were all about 200 nm in each fraction (Figure 1A), and representative particle size distribution of three methods are shown (Figure 1B). For the yield of particles, the pEVs eluted with a peak at fractions 5 and 6 in UC-SEC method and with a peak at the density of 1.093–1.111 g/mL in UC-DGU method (Figure 1C). It should be noted that these counted particles by the NTA are not necessarily real EVs. The protein content of the eluted fractions in UC-SEC method was with a peak at fractions 10 to 13, which was dramatically different from the results of the particle counts peak. The similar results were also observed in UC-DGU method (Figure 1D). The results suggested that UC-SEC and UC-DGU may successfully remove the contaminated proteins. The ratio of the number of particles to the protein content (particle/protein ratio) has previously been suggested to be an adequate marker of sEV purity (Webber and Clayton, 2013). Through the purity analysis, the eluted fractions in UC-SEC method were with a particle/protein ratio peak at fractions 5 and 6, and with a ratio peak at the density of 1.093–1.111 g/mL in UC-DGU method. Therefore, these fractions were subsequently referred to as “SEC-peak” and “DGU-peak” which with the high purity pEVs. The results of purity analysis showed that UC-SEC method produced the highest yield of high-purity pEVs, followed by UC method, and UC-DGU performed the lowest efficiency (Figure 1E).

### Morphological characteristics and EVs markers of pEVs isolated by different methods

**FIGURE 2:**
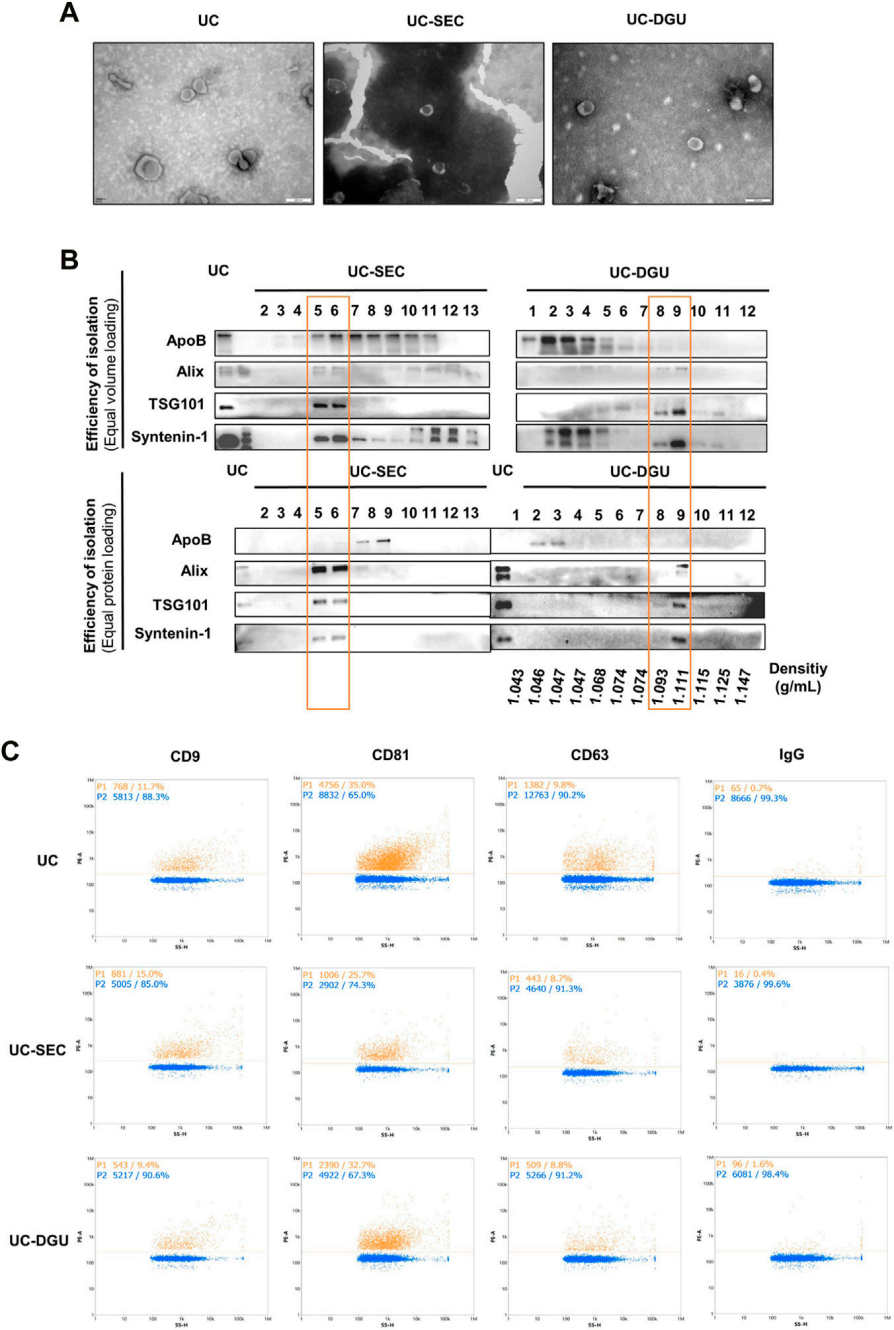
Characterization of pEVs. **(A)** The morphological characteristics of pEVs were detected by TEM. **(B)** The EVs markers (Alix, TSG101 and Syntenin-1) and non-EVs marker (ApoB) were detected by Western blotting. 20 uL sample or 20 μg protein from each fraction was loaded. **(C)** The pEVs were fluorescently labeled with PE-conjugated EVs mAbs (CD9, CD63 and CD81), and the positive ratio was detected by nFCM measurement.

The morphological characteristics of pEVs isolated by UC, UC-SEC and UC-DGU were observed by TEM. As the result, cup-shaped particles with different sizes were observed in all three methods Cup-shaped structures indicate intact bilipid membranes. The pEVs samples prepared by UC and UC-SEC were generally with a relatively clear background. However, in the UC-DGU method, the cup-shaped particles were surrounded by a few non-EV particles (For example, lipids and protein aggregates) (Figure 2A).

To assess the peak fraction and determine the efficiency of SEC and DGU method, first, equal volumes of UC, SEC and DGU fractions were analyzed by Western blotting. Meanwhile, the purity of each fraction was tested by loading an equal amount of protein following protein quantification. As a result of higher expression of specific EVs markers indicating a higher concentration of ‘true EVs’, we employed the protein markers suggested by MISEV2018 to estimate the purity of pEVs. Three EVs markers (Alix, TSG101 and Syntenin-1) were used as positive makers to identify the presence of EVs and ApoB was tested as a non-EVs marker, which was the common contaminated protein in PE sample. In UC method, all three markers were well detected and the expression of ApoB was lower. When loading equal volumes of fractions, the peak fractions of EVs markers appeared at fractions 5, 6 and 10 to 13 in SEC method and appeared at fractions 8, 9 and 2 to 5 in DGU method (Figure 2B), which were consistent with the result of particle concentration and protein yield. However, when loading the same amount of protein from each sample, the three EV markers are enriched in fractions 5 and 6, while ApoB is enriched in fractions 7 and 8 in SEC method, and the three EVs markers are enriched in fractions 9, while ApoB is enriched in fractions 2 and 3 in DGU method (Figure 2B). These results showed that the majority of Alix, TSG101 and Syntenin-1 appeared at different fractions of ApoB, which indicates that the separation methods of SEC and DGU was effective.

In addition, the membrane surface marker of EVs (CD9, CD81 and CD63) were detected by nFCM. All three membrane markers of EVs were detected in samples from each method, but the expression level of each marker was different. The percentages of CD81 positive particles were the highest, while the positive ratio of CD63 and CD9 were lower (Figure 2C), and there was the same trend in all three methods. Altogether, these results illustrated that pEVs were successfully collected and purified by all three methods.

### Proteomic profiling of pEVs isolated by different methods

**FIGURE 3:**
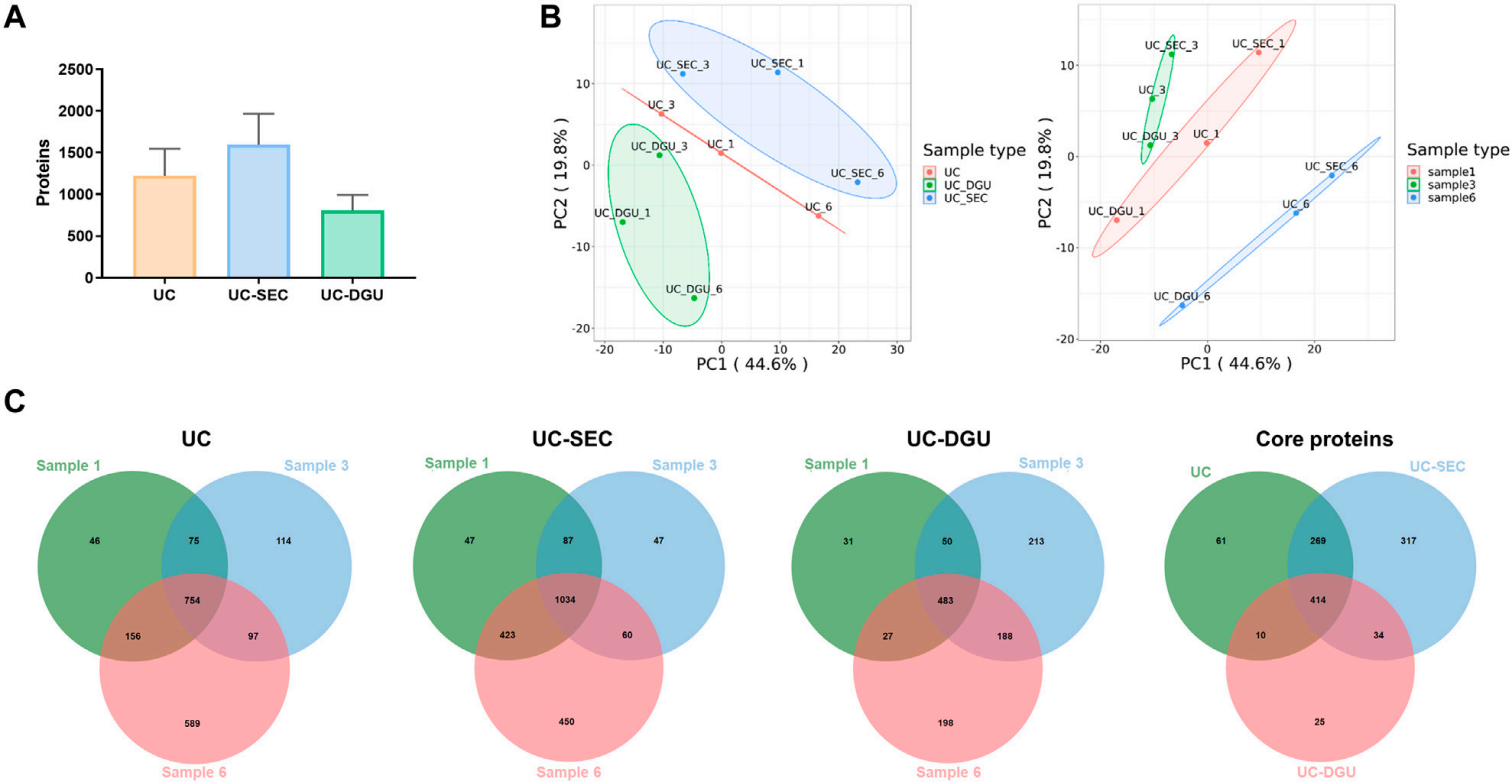
Proteomic analysis of pEVs isolated by three methods. **(A)** The total protein quantity of pEVs isolated by three methods were determined by mass spectrometry-based analysis. **(B)** Principal component analysis (PCA) of pEVs proteins for three methods and three samples. **(C)** Venn diagram for the overlapped and unique proteins of pEVs isolated by the three methods.

Since there is no way to completely isolate pure EVs, proteomics analysis may help to illustrate the quality of EVs isolation holistically. To explore the impact of different isolation methods on Proteomic Profiling of pEVs, the label-free quantitative proteomics was used. Quantitative proteomics results showed that there were significant differences in the number of proteins identified by the three isolation methods. The most quantity of proteins were identified by UC-SEC (1595 ± 213.3), followed by UC (1222 ± 186.9) and UC-DGU (807 ± 108.6) (Figure 3A). The methodological repeatability and biological repeatability of samples were assessed by principal component analysis (PCA). The results showed that the pEVs isolated by different method were clustered well, especially the pEVs for UC-SEC method was clustered away from pEVs for UC-DGU. Similarly, the samples from different individuals also clustered well (Figure 3B). These results indicate that the proteomic profiling of pEVs isolated by each method was remarkable different, and the repeatability of each method and each sample was well. The Venn diagram showed the overlap of the identified proteins from all samples. There were 754 proteins identified in all samples by UC method and were defined as “core proteins in UC”. Similarly, there were 1034 core proteins in UC-SEC and 483 core proteins in UC-DGU. Among these, 414 proteins were commonly identified in pEVs isolated by all three methods, and the 414 proteins were defined as “core proteins” in the subsequent study (Figure 3C). The number of uniquely identified proteins was 61 for UC, 317 for UC-SEC and 25 for the UC-DGU method. The results showed that, consistent with the number of total proteins identified in each method, most core proteins were identified by UC-SEC and fewest core proteins were detected by the UC-DGU method. It’s probably due to the presence of high-abundance proteins in UC-DGU fractions, which prevented low-abundance proteins to be identified.

### pEVs was successfully isolated by all three methods

**FIGURE 4:**
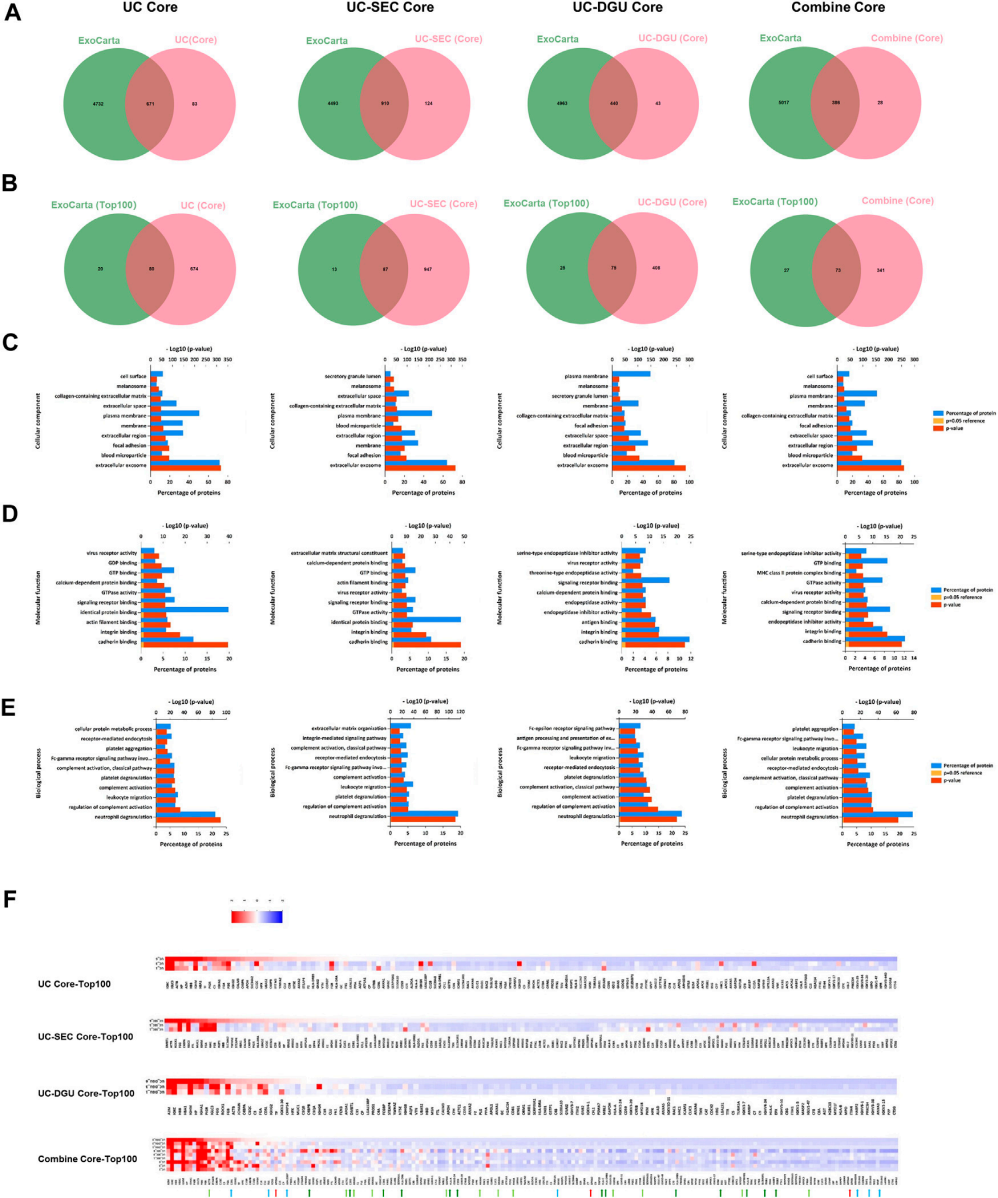
The characteristic of identified core proteins by three methods. **(A)** The identified core pEVs proteins by each method were compared with the proteins described in ExoCarta database. **(B)** The identified core pEVs proteins by each method were compared with the top 100 proteins listed in ExoCarta database. **(C–E)** The identified core pEVs proteins by each method were annotated by GO annotation analysis, and the cellular components, molecular functions and biological processes were categorized according to *p-value*. **(F)** The top 100 proteins of identified core pEVs proteins by each method were matched with EVs markers suggested by MISEV 2018.

To further verify the efficiency of the three methods for separating pEVs, we compared identified pEVs proteins with the proteins described in ExoCarta database, which is a web-based compendium of known EVs cargo (Mathivanan et al., 2012; Keerthikumar et al., 2016). As a result, there are 88.99% (671/754) of the core proteins in UC were matched with the ExoCarta database, and 88.01% (910/1034) and 91.10% (440/483) in UC-SEC and UC-DGU method respectively. In addition, there are 93.24% of the core proteins (386/414) identified by all three methods are ExoCarta reported EVs proteins (Figure 4A). Furthermore, there were 73% of core proteins identified by all three methods annotated in the top 100 protein list of ExoCarta, of which the core proteins identified by the UC-SEC method account for the highest proportion of ExoCarta Top100 proteins (87%) (Figure 4B). Besides, GO annotation analysis was used to categorize the proteins identified by three isolation methods as cellular components, molecular functions and biological processes (Figures 4C–E). The top 10 statistically significant items are listed in sequence. The results showed that most of the pEVs proteins identified by the three methods were localized in “extracellular exosome” (Figure 4C). These results indicated that pEVs could be successfully isolated by all three methods.

In addition, we defined the proteins that were only identified by one method as “unique proteins” and the unique proteins in each method were also compared with the proteins in ExoCarta database. Interestingly, about 80% of the unique proteins identified by each method were also matched with the reported EV proteins in ExoCarta (Supplementary Figure S1A), however, there were 5 proteins only identified by UC-SEC method annotated in the top 100 protein list of ExoCarta (Supplementary Figure S1B). GO annotation analysis was also used to categorize the unique proteins identified by three methods (Supplementary Figures S1C–E). The results showed that UC-UC and UC-SEC unique proteins are also enriched in “extracellular exosome”, while UC-DGU unique proteins are enriched in “proteasome core complex” (Supplementary Figure S1C). These results suggested that the pEVs isolated by UC-SEC method identified the maximum quantity and largest proportion of EVs proteins.

In order to study the significance of highly expressed proteins identified in each method, the top 100 proteins of each method in all three samples were listed (Figure 4F). the protein markers suggested by MISEV2018 were employed to estimate the purity of pEVs. As shown, these colored arrows represent several categories of proteins described in MISEV2018 that demonstrate EVs purity. The dark green arrow represents transmembrane or GPI-anchored proteins associated with the plasma membrane and/or endosomes, the light green arrow represents cytosolic proteins recovered in EVs, the blue arrow represents secreted proteins recovered with EVs and the red arrow represents major components of non-EV co-isolated structures. The results showed that compared with the protein markers which suggested to validate the presence of EVs in MISEV2018 guidelines, many of the top 100 high expression proteins in each method were matched with the guidelines.

### Analysis of potential efficient protein markers of pEVs

**FIGURE 5:**
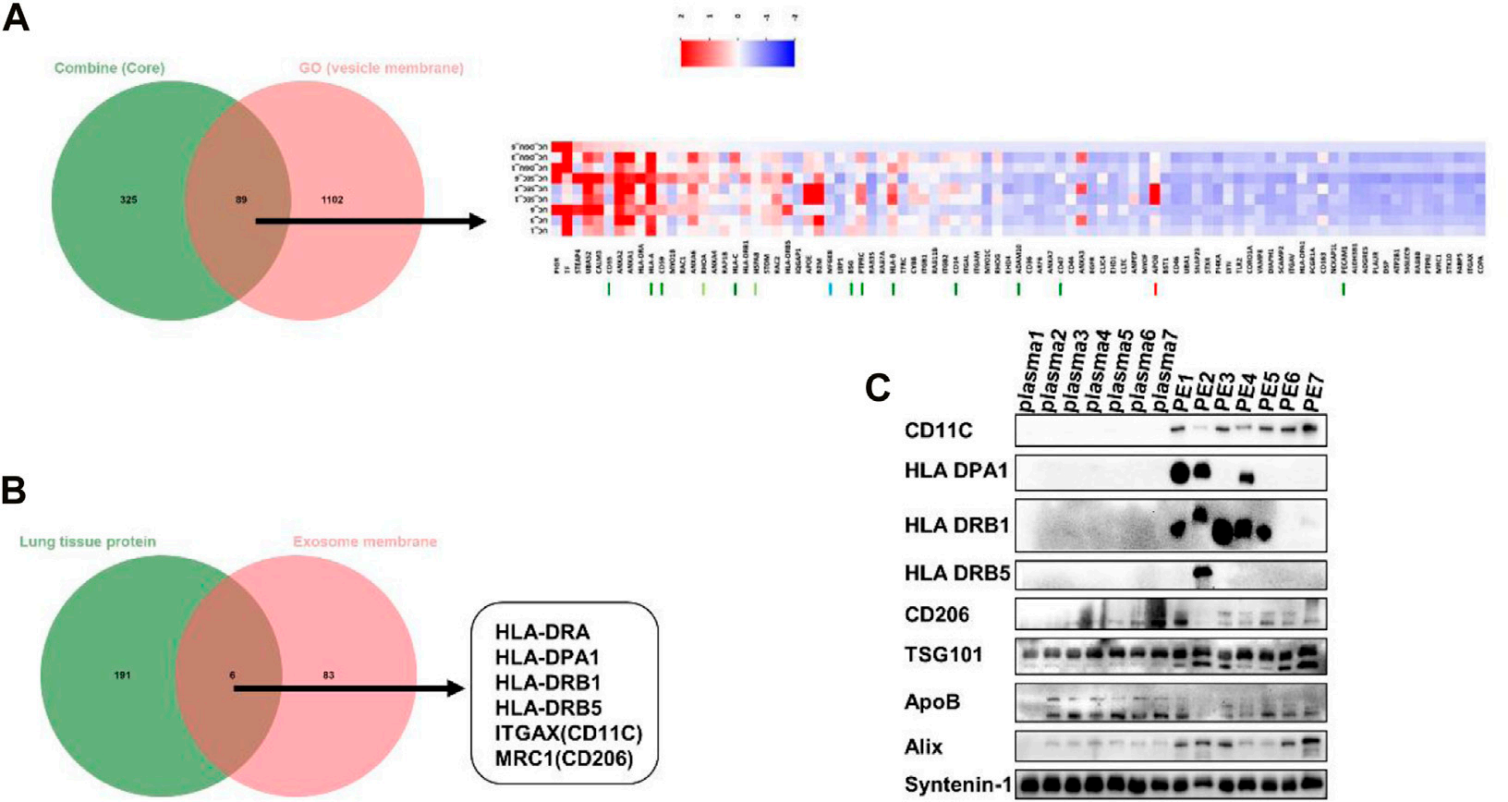
The potential specific protein markers of pEVs. **(A)** The three methods shared core proteins and vesicle membrane proteins were overlapped by Venn analysis. **(B)** The pEVs membrane proteins and lung tissue specific proteins were overlapped by Venn analysis, and six unique pEVs membrane markers were found (HLA-DPA1, HLA-DRA, HLA-DRB1, HLA-DRB5, CD11C and CD206). **(C)** The expression level of the six protein markers was verified in EVs from PE and plasma by Western blotting.

To screen the potential efficient protein markers of pEVs, especially the membrane markers which are easy to detect, the three methods shared core proteins and vesicle membrane protein annotated in GO were overlapped by Venn analysis. The results showed that there were 89 vesicle membrane markers were found and many of them were suggested by MISEV 2018 (Figure 5A). Further, to screen the specific membrane markers of pEVs, the 89 vesicle membrane markers and lung tissue specific proteins were overlapped. As a result, six potential EVs membrane markers specific to the pEVs were found: HLA-DPA1, HLA-DRA, HLA-DRB1, HLA-DRB5, ITGAX (CD11C) and MRC1(CD206) (Figure 5B). To further validate these results, the EVs derived from pleural fluid and plasma of 7 patients were isolated and analyzed by Western blotting.

For reference, we characterized plasma derived extracellular vesicles (Supplementary Figure S2). The EVs markers TSG101, Alix and syntenin-1 were used as the internal standard and positive control. The results showed that the levels of CD11C, HLA DPA1 and HLA DRB1 were significantly higher in pEVs compared with plasma derived EVs, which was consistent with the results of proteomics (Figure 5C). The results suggested that CD11C, HLA DPA1 and HLA DRB1 may be the potential efficient protein markers of pEVs. In order to verify whether these protein markers are specific markers of PE sample or just high expressed in lung cancer, we selected patients with bacterial pneumonia with benign pleural effusion as controls. pEVs from BPE and MPE were isolated and analyzed by Western blotting. The results showed that CD11C, HLA DPA1 and HLA DRB1 were still highly expressed in BPE and MPE (Supplementary Figure S3).

## Discussion

Pleural effusion-derived EVs are considered to be valuable biomarkers for early molecular diagnosis of lung cancer, tuberculosis and other diseases (Wang et al., 2017; Luo et al., 2020; Javadi et al., 2021; Kato et al., 2021). The effective methods for isolating high-purity EVs remain a major challenge, which limits the application of EVs in early disease diagnosis and clinical translation.

In this study, we explored the effective method to isolate pEVs. Our findings demonstrate that all three methods (UC-UC, UC-SEC and UC-DGU) could successfully separate EVs from human pleural effusion. However, the results of particles/protein, TEM and Western blotting showed that with comprehensive considering of yield and purity of pEVs, UC-SEC is a more efficient method. When the yield was assessed by NTA, which was based on the number of particles, the non-EV particles such as protein aggregates may be detected and therefore the number of real EVs may be overestimated. When the yield was assessed by nFCM, which used fluorescent antibodies and UC has performed again before the measurement, the yield of real EVs may be underestimated.

The positive ratio of tetraspanins of EVs (CD9, CD81 and CD63) were different in the same sample, in which CD81 with the highest positive rate and CD63 with the lowest. It may be because the commonly used EVs biomarkers are heterogeneous and are not universally present in EVs from different samples (Kugeratski et al., 2021). The results suggested that it is inappropriate to use CD9 and CD63 as the pEVs markers.

In addition, proteomic analysis revealed that pEVs isolated by UC-SEC identified more total proteins and more kinds of EVs-associated proteins. When comparing these identified core proteins by each method with EVs proteins in the Exocarta database, the results showed that about 90% of core proteins in each method were matched in Exocarta and notably, there were 28 common core proteins were not reported by Exocarta, and these proteins may be new EVs markers that not reported. When compared these identified unique proteins by each method with Exocarta proteins list, there were about 80% of unique proteins in each method were matched in Exocarta and interestingly, 5 unique proteins in UC-SEC method matched the top 100 proteins list in Exocarta, and these proteins may be the specific pEVs proteins in UC-SEC method. When annotating the core proteins and unique proteins identified by each method in GO database, the results showed that the core proteins identified by all three methods were mainly localized in “extracellular exosome”. The unique proteins identified by UC-UC and UC-SEC are also enriched in “extracellular exosome”, while the unique proteins in UC-DGU are enriched in “proteasome core complex”. Although pEVs isolated by UC-DGU have the highest protein content, LC-MS analysis identified the fewest protein species in UC-DGU. The results indicated that the pEVs isolated by UC-DGU were impurities, and the high-abundance proteins masked mass spectrometry detection of these low-abundance proteins, while the UC-SEC method could get pEVs with higher purity and identify more proteins by LC-MS analysis. In summary, compared with UC and UC-DGU, UC-SEC could obtain pEVs with higher purities and detect more proteins in mass spectrometry, which is more conducive to downstream research. In addition, SEC method takes only 30 min, which is significantly faster than UC (70 min) and DGU (18 h). There are already many research teams working to address this difficulty (Stranska et al., 2018; Takov et al., 2019; Guan et al., 2020; Jalaludin et al., 2021), however, different samples and different isolation methods affect the purity and yield of EVs, so there is still no gold standard for isolation.

The results of GO-MF analysis showed that the core proteins in all three methods are enriched in cadherin binding, which may indicate that tumor cells transfer cadherin through EVs. In tumors, the loss of cadherin (especially E-cadherin) can lead to reduced adhesion between tumor cells, contribute to epithelial-interstitial transformation, and promote the ability of tumor cells to invade and metastasize (Kaszak et al., 2020). Studies have shown that the rich expression of E-cadherin in ascites derived EVs in ovarian cancer patients is related to malignant ascites formation and widespread peritoneal spread, and soluble E-cadherin promotes tumor angiogenesis and localization to the EVs surface (Tang et al., 2018), which is similar to our results. The results of GO-BP analysis showed that the core proteins in all three methods are enriched in neutrophil degranulation, some researchers have reported that neutrophils play a role in carcinogenesis in various ways, including releasing extracellular traps to promote tumor metastasis and enhancing the malignant potential of circulating tumor cells (Yang et al., 2020). There is some evidence to suggest that neutrophils in tumors could suppress T-cell immune responses and make immune checkpoint inhibitors ineffective against tumors (Singhal et al., 2016). It has been demonstrated that tumor-associated neutrophil (TAN) could promote tumor cell proliferation, extravasation and migration. TAN could release particulate components, such as elastase, to promote cancer cell proliferation and invasion (Dou and Fang, 2021; Peña-Romero and Orenes-Piñero, 2022). The results indicated that the pEVs proteins could be developed as diagnosis and prognosis biomarkers of lung cancer.

After overlapping the core proteins, vesicle membrane proteins and lung tissue specific proteins, there were 6 proteins were found as specific pEVs membrane markers, which are HLA-DPA1, HLA-DRA, HLA-DRB1, HLA-DRB5, ITGAX (CD11C) and MRC1(CD206). Comparing the expression level of the 6 proteins in EVs derived from plasma and pleural effusion, CD11C, HLA DPA1 and HLA DRB1 could be considered as the efficient pEVs markers. Compare the expression level of the 6 proteins in EVs derived from BPE and MPE, CD11C, HLA DPA1 and HLA DRB1 could also be considered as the efficient pEVs markers. This suggests that these three markers may be specific markers of pleural effusion but not only high expressed in MPE. However, this result need to be confirmed by enlarging the sample size.

Tumor-associated macrophages (TAMs) are activated macrophages associated with tumor progression in various cancers such as lung cancer, breast cancer, and ovarian cancer (Qiu et al., 2018; Nowak and Klink, 2020; Song et al., 2020; Tan et al., 2021). Generally, macrophages are generally classified as M0 (resting macrophages), M1 (classically activated), and M2 (alternately activated) (Li Y. et al., 2021). In our study, M1 markers HLA DR, HLA DP and CD11C were expressed specifically, while M2 markers CD206 were expressed unspecifically. In a recent study, the lung tissue of cancer patients possesses an enriched population of macrophages characterized by EV secretion Additionally, the class II MHC protein, HLA-DR was expressed on 40% of EVs secreted from M1-like human monocyte-derived macrophages, which was twice as frequent as M0-like and M2-like EVs (Dechantsreiter et al., 2022). This is consistent with our findings. From this, it can be seen that macrophages towards a proinflammatory mainly polarization and pEVs in lung cancer patients may play an anti-tumor immunity role. The special role of these molecules in the formation or the recurrence of pleural effusion remains an enigma.

The pleura belongs to anatomically adjacent structures of the lung (Miserocchi, 1997; Wang et al., 2018). Due to this close relationship, PE is highly enriched with EVs that originate from lung lesions and contain numerous components released by cancerous cells (Haggadone and Peters-Golden, 2018). Therefore, there exists considerable potential for pEVs to serve as a valuable tool for the diagnosis of pulmonary diseases. In the present study, we systematically compared three methods for isolation of PEVs to determine the most appropriate separation scheme. On the basis of this study, we can further isolate and purify PE derived EVs from different lung diseases, and explore the contents of pEVs such as DNA, RNA, proteins and metabolite, so as to find more appropriate biomarkers to play a greater role in the early diagnosis and clinical treatment of lung diseases.

There were some limitations in this study. First, the sample size in the study was small, and some results need to be further verified. Second, the present study focuses on finding an efficient method for isolation of pEVs, so only lung cancer is included in MPE and only bacterial pneumonia is included in BPE. In future research, the efficient method will be used to isolate pEVs in more diseases to identify BPE and MPE to identify the novel lung disease markers.

In summary, UC-SEC method is suitable for separating EVs from pleural effusion and for downstream proteomic analysis. Moreover, CD11C, HLA DPA1 and HLA DRB1 may be the potential specific markers for EVs from pleural effusion.

## Data Availability

The original contributions presented in the study are included in the article/Supplementary Material, further inquiries can be directed to the corresponding authors.

## Supplementary material

SUPPLEMENTARY FIGURE S1 The characteristic of identified core proteins by three methods. **(A)** The identified unique pEVs proteins by each method were compared with the proteins described in ExoCarta database. **(B)** The identified unique pEVs proteins by each method were compared with the top 100 proteins listed in ExoCarta database. **(C–E)** The identified unique pEVs proteins by each method were annoted by GO annotated analysis, and the cellular components, molecular functions and biological processes were categorized according to *p*-value.

SUPPLEMENTARY FIGURE S2 The characteristic of EVs separated from plasma. **(A)** Representative particle size distribution plots of EVs separated from plasma were measured using NTA. **(B)** The morphological characteristics of EVs separated from plasma were detected by TEM. **(C)** The EVs separated from plasma were fluorescently labeled with PE-conjugated EVs mAbs (CD9, CD63 and CD81), and the positive ratio was detected by nFCM measurement.

SUPPLEMENTARY FIGURE S3 The expression level of the six protein markers (HLA-DPA1, HLA-DRA, HLA-DRB1, HLA-DRB5, CD11C and CD206) were verified in EVs from Benign pleural effusion (BPE) and Malignant pleural effusion (MPE).

SUPPLEMENTARY TABLE S1 Basic patient information.
