# Supplementary figures and images for: Comparison of proteomic landscape of extracellular vesicles in pleural effusions isolated by three strategies
Source: Front Bioeng Biotechnol. 2023 Apr 12;11:1108952. doi: 10.3389/fbioe.2023.1108952 (PMC10130534; doi:10.3389/fbioe.2023.1108952)

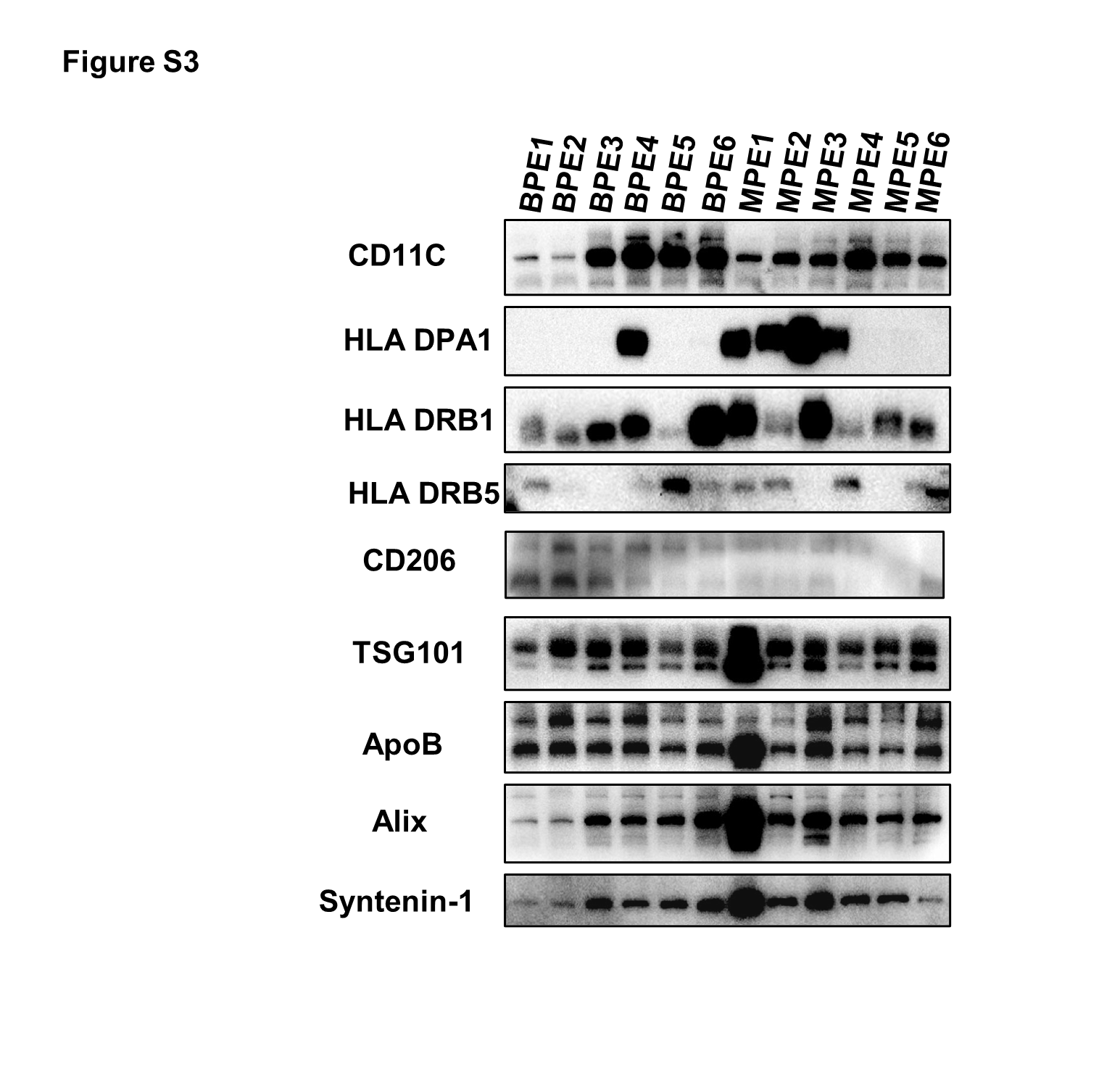

Supplement: Supplementary file 1 [file Image3.TIF]

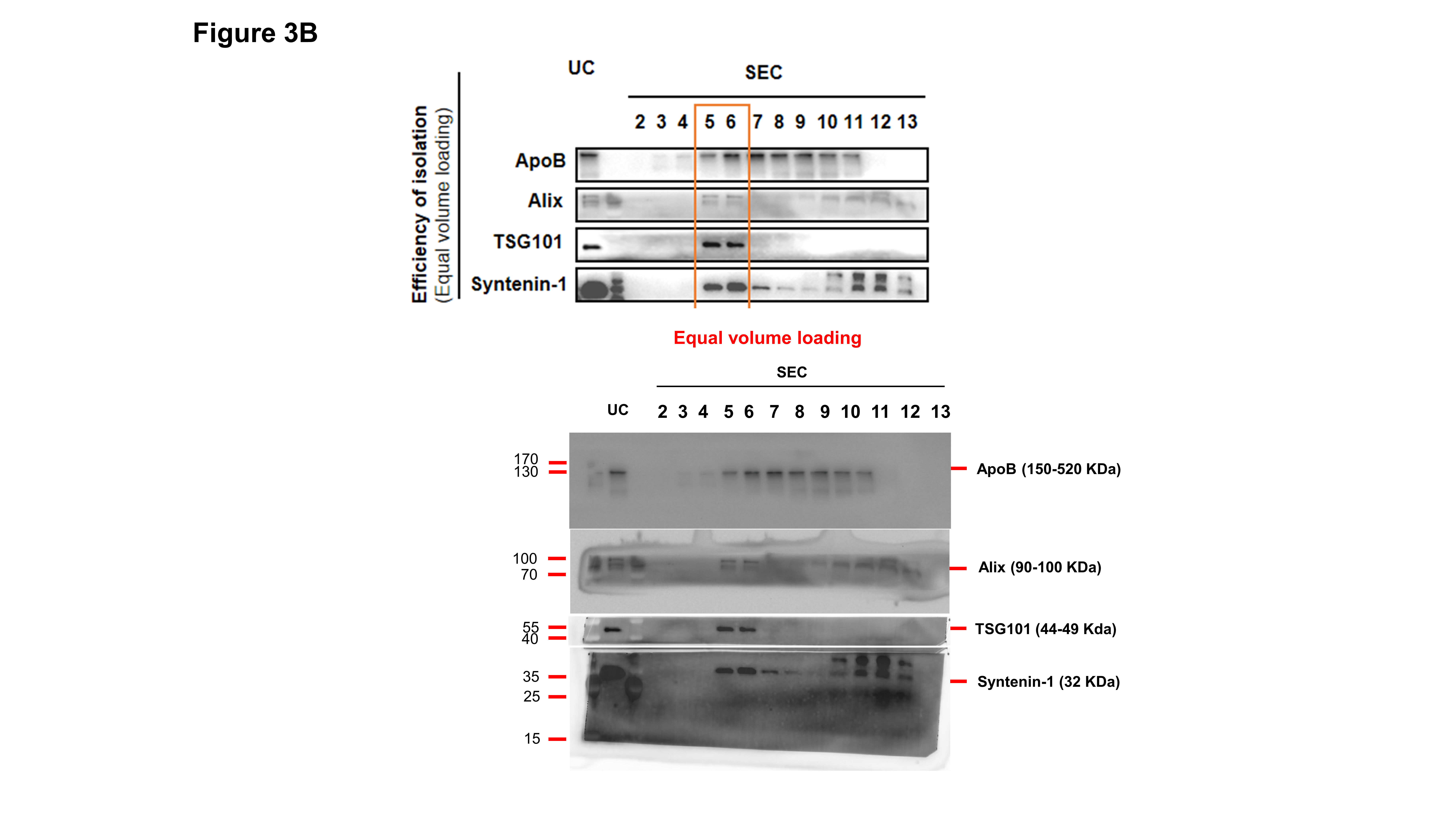

Supplement: Supplementary file 2 [file DataSheet1.ZIP › Original Images for Blots/╗├╡╞╞1⁄41.TIF]

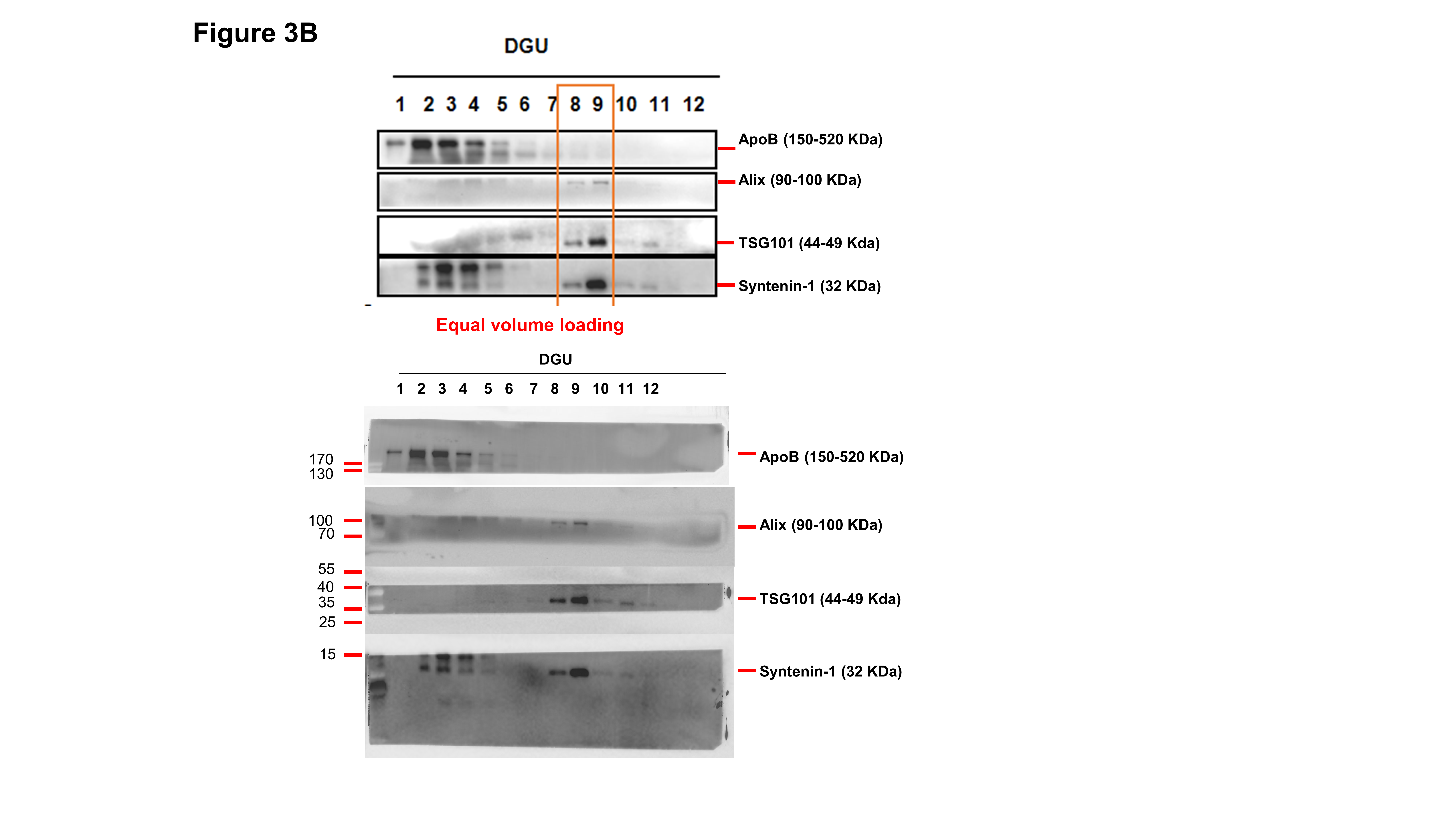

Supplement: Supplementary file 2 [file DataSheet1.ZIP › Original Images for Blots/╗├╡╞╞1⁄42.TIF]

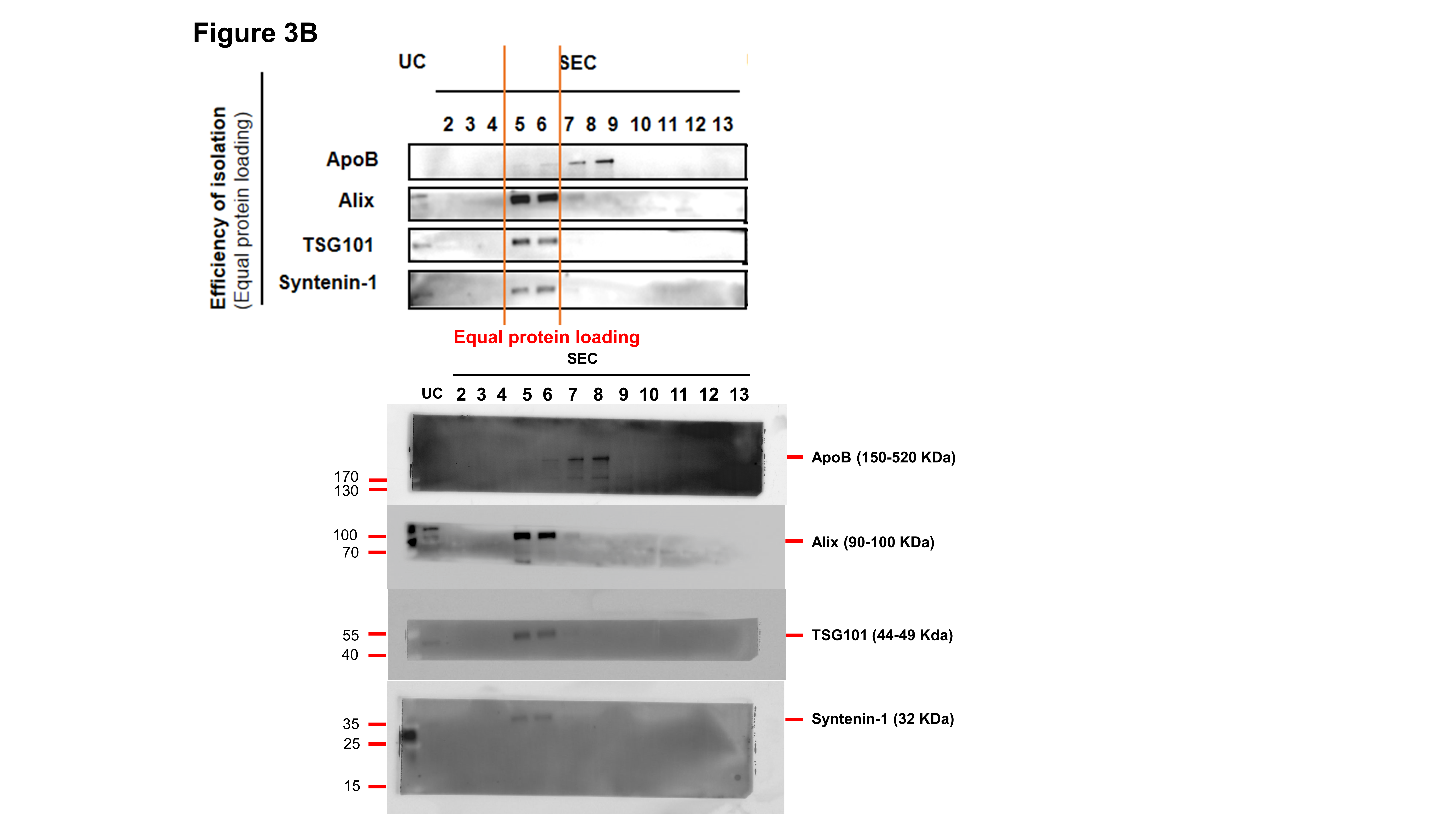

Supplement: Supplementary file 2 [file DataSheet1.ZIP › Original Images for Blots/╗├╡╞╞1⁄43.TIF]

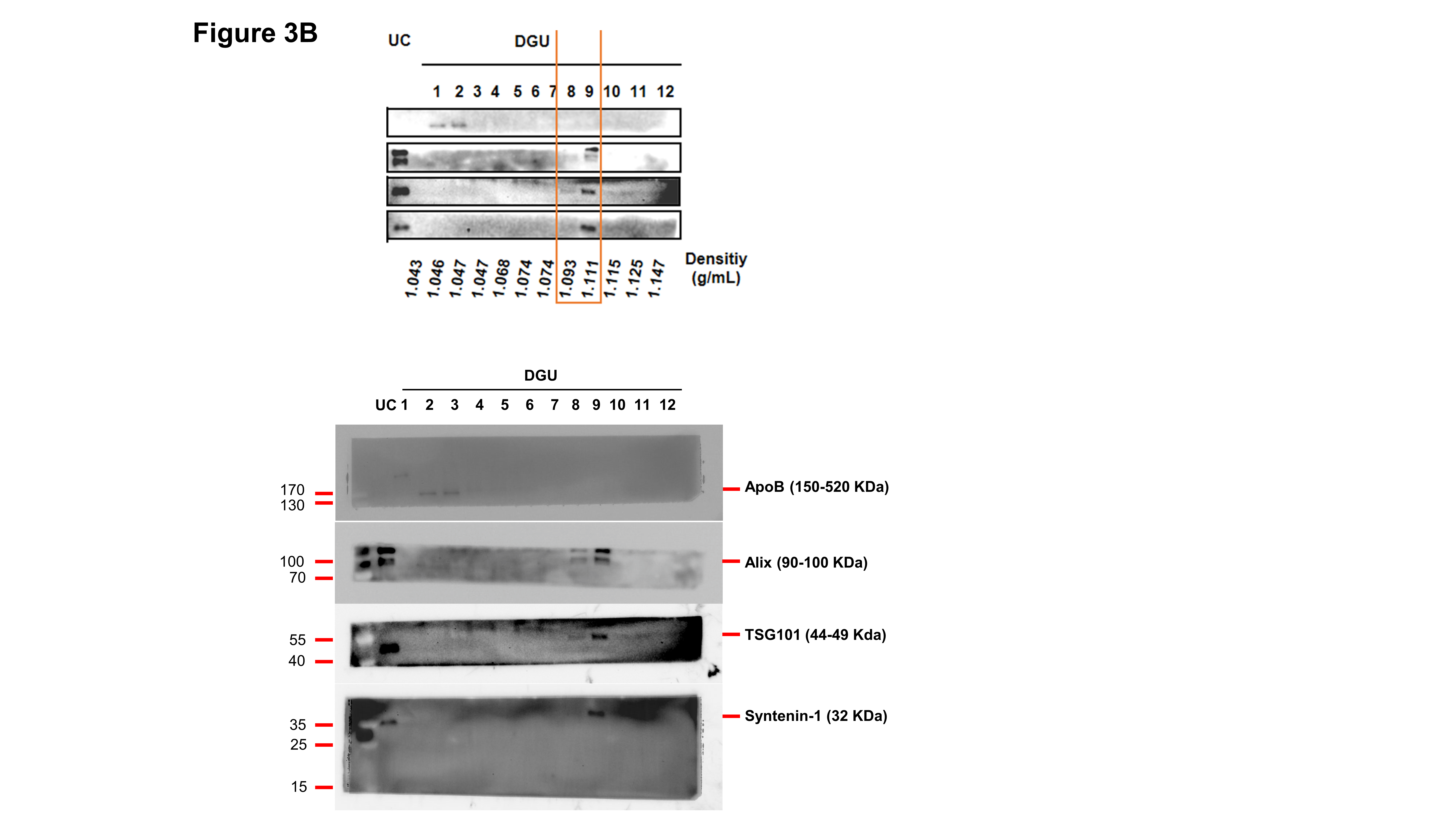

Supplement: Supplementary file 2 [file DataSheet1.ZIP › Original Images for Blots/╗├╡╞╞1⁄44.TIF]

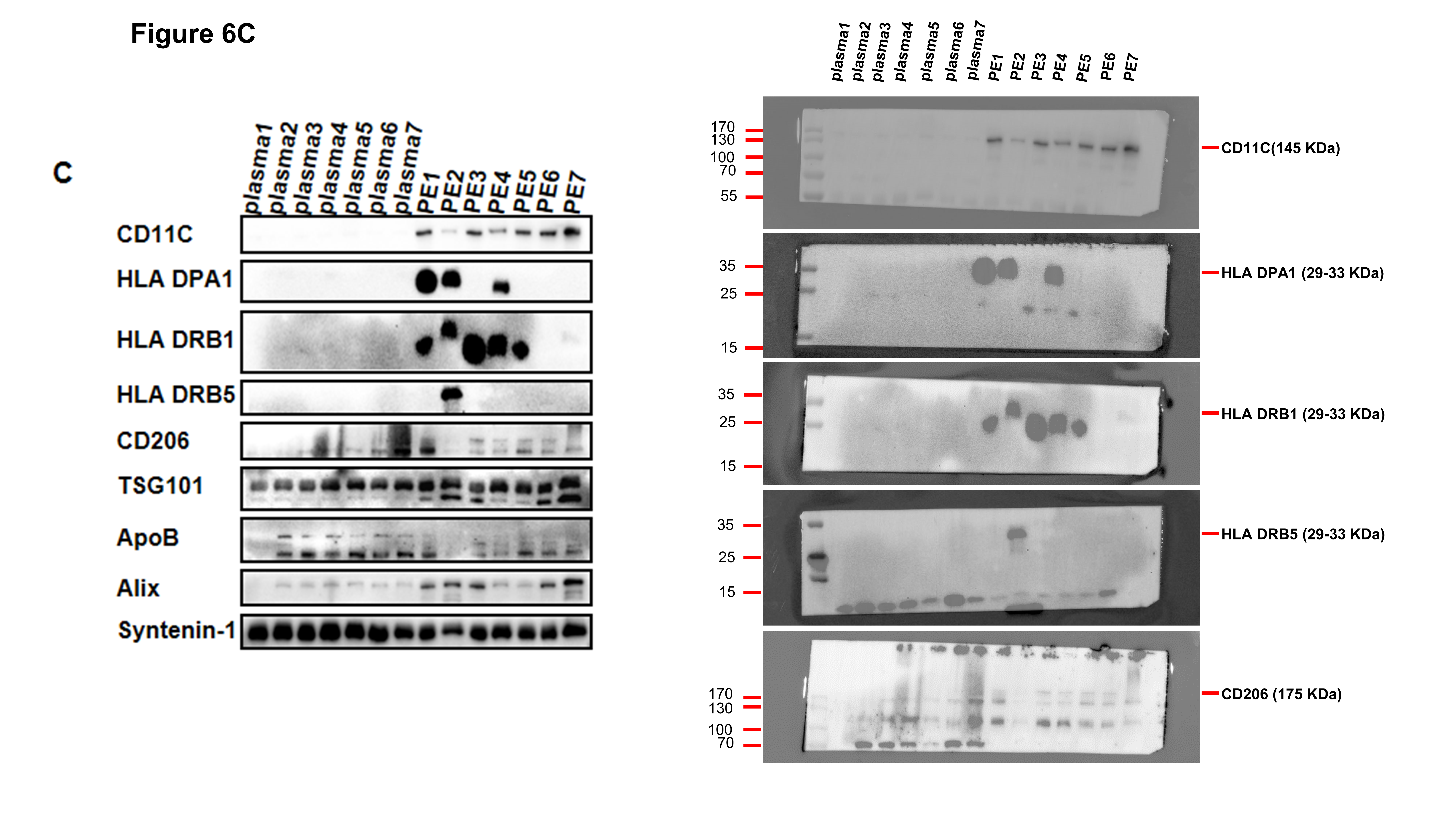

Supplement: Supplementary file 2 [file DataSheet1.ZIP › Original Images for Blots/╗├╡╞╞1⁄45.TIF]

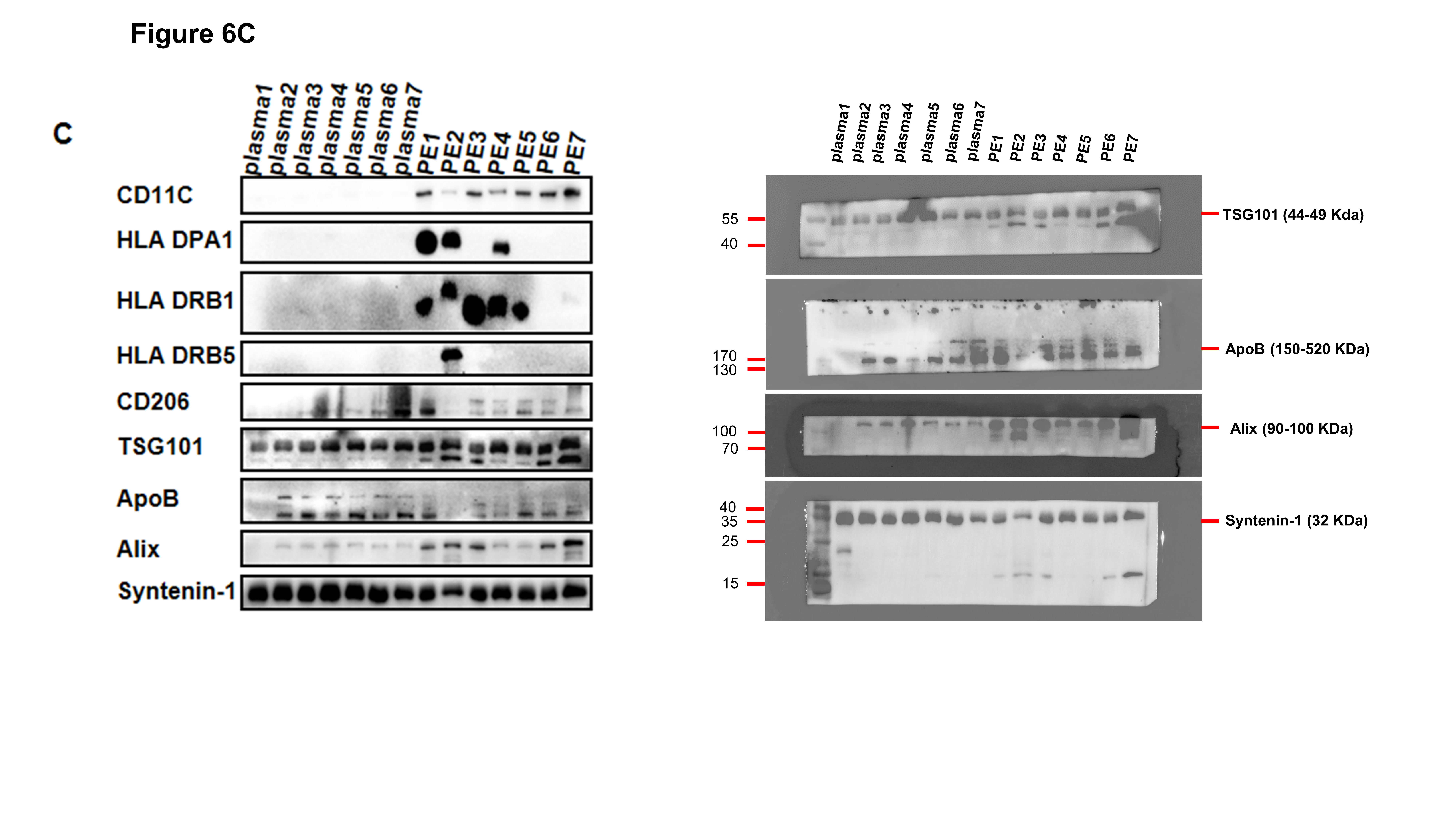

Supplement: Supplementary file 2 [file DataSheet1.ZIP › Original Images for Blots/╗├╡╞╞1⁄46.TIF]

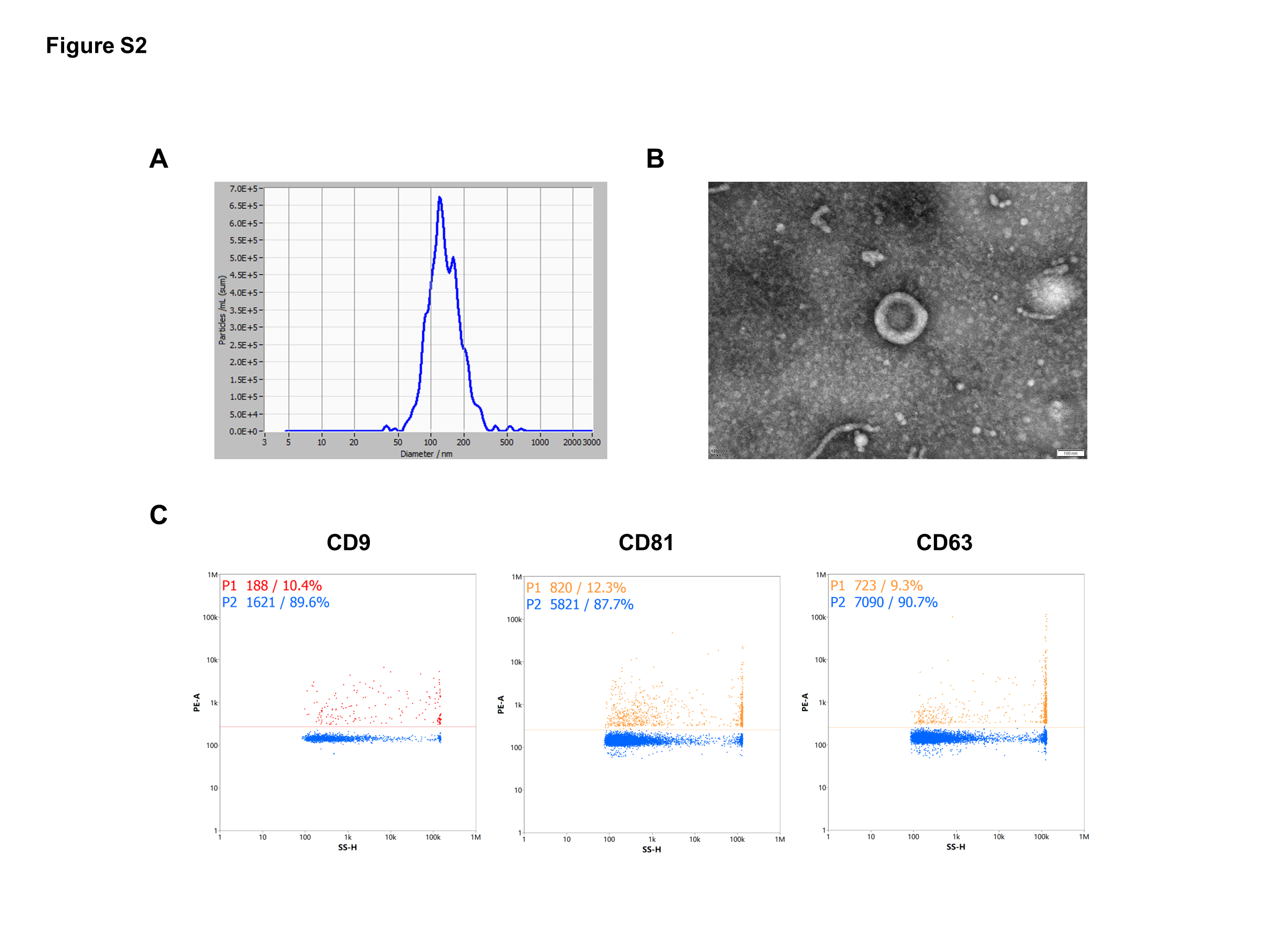

Supplement: Supplementary file 3 [file Image2.TIF]

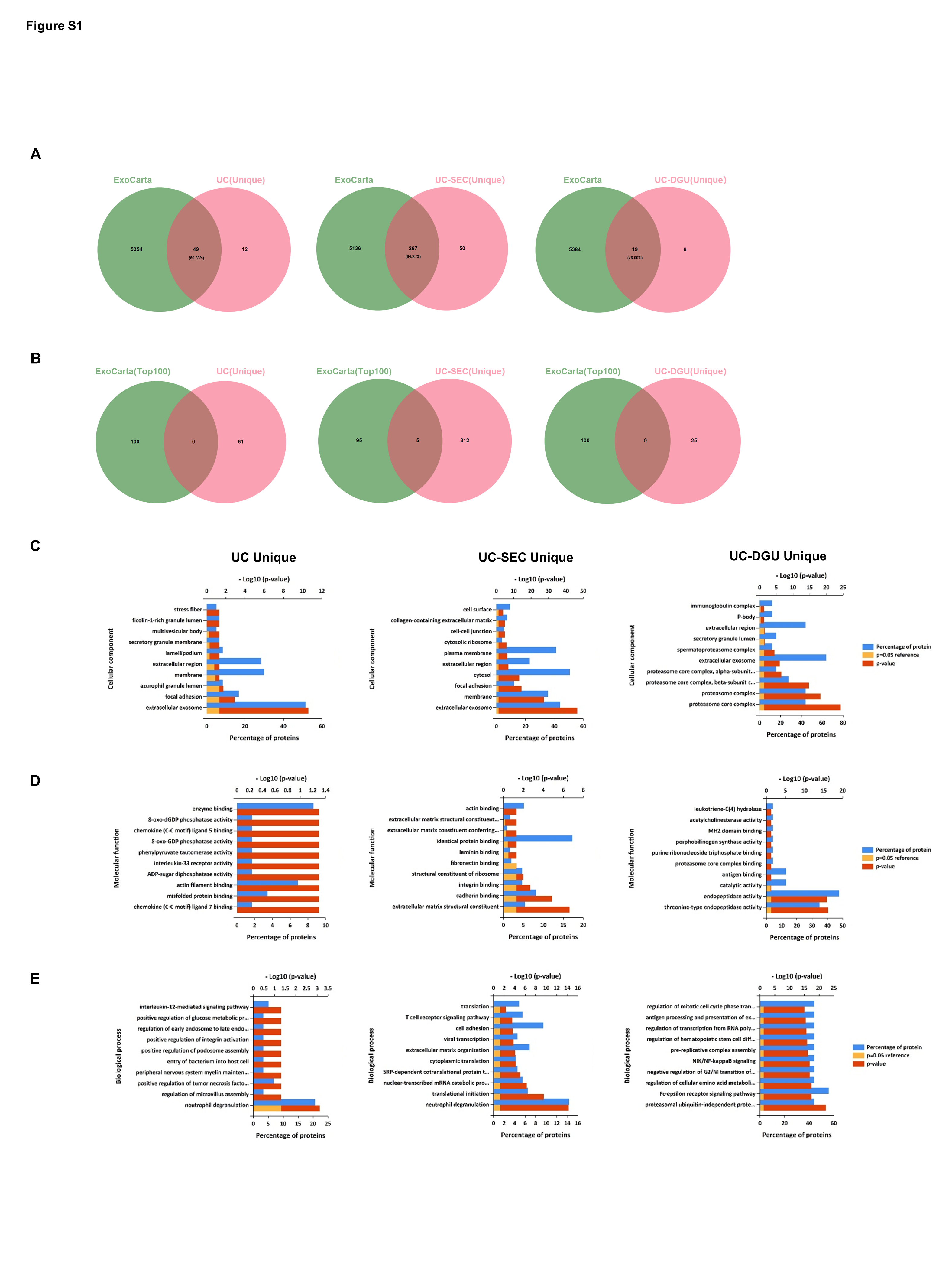

Supplement: Supplementary file 4 [file Image1.TIF]
